# Efficacy of *Poria cocos* and *Alismatis rhizoma* against diet-induced hyperlipidemia in rats based on transcriptome sequencing analysis

**DOI:** 10.1038/s41598-023-43954-6

**Published:** 2023-10-15

**Authors:** Xiaowen Zhou, Jingbiao Luo, Shuxian Lin, Yaxin Wang, Zhenqian Yan, Qi Ren, Xiaoqi Liu, Xiantao Li

**Affiliations:** 1 The Second School of Clinical Medicine, Guangzhou University of Chinese Medicine, Guangzhou, 510405 China; 2 Laboratory of TCM Syndrome Essence and Objectification, School of Basic Medical Sciences, Guangzhou University of Chinese Medicine, No. 232, East Waihuan Road, Guangzhou Higher Education Mega Centre, Panyu District, Guangzhou City, 510006 China; 3 Department of Rheumatology and Immunology, Southwest Hospital, Army Medical University, Chongqing, 400038 China; 4 NanoFCM Inc, Xiamen, 361006 China

**Keywords:** Drug discovery, Computational biology and bioinformatics, Functional clustering, High-throughput screening, Diseases, Endocrine system and metabolic diseases

## Abstract

Hyperlipidemia, a common metabolic disease, is a risk factor for cardiovascular diseases, *Poria cocos* (PC) and *Alismatis rhizoma* (AR) serve as a potential treatment. A systematic approach based on transcriptome sequencing analysis and bioinformatics methods was developed to explore the synergistic effects of PC–AR and identify major compounds and potential targets. The phenotypic characteristics results indicated that the high dose (4.54 g/kg) of PC–AR reduced total cholesterol (TC), elevated high-density lipoprotein cholesterol (HDL-C) levels, and improved hepatocyte morphology, as assessed via hematoxylin and eosin (H&E) staining. Transcriptomic profiling processing results combined with GO enrichment analysis to identify the overlapping genes were associated with inflammatory responses. The cytokine-cytokine receptor interaction pathway was found as a potential key pathway using geneset enrichment analysis. Core enrichment targets were selected according to the PC–AR's fold change versus the model. Real-time quantitative PCR analysis validated that PC–AR significantly downregulated the expression of Cxcl10, Ccl2, Ccl4, Cd40 and Il-1β mRNA (*P* < 0.05). Molecular docking analysis revealed the significant compounds of PC–AR and the potential binding patterns of the critical compounds and targets. This study provides further evidence that the therapeutic effects of PC–AR on hyperlipidemia in rats through the regulation of inflammation-related targets.

## Introduction

Hyperlipidemia (HLP), a lipid metabolism disorder, is an umbrella term for the abnormal elevation of total cholesterol (TC), triglycerides (TG) and low-density lipoprotein cholesterol (LDL-C) or a reduction in high-density lipoprotein cholesterol (HDL-C)^1^. HLP contributes to the risk of arteriosclerosis, stroke, diabetes, and renal failure^2,3^, and has begun to affect younger generations, primarily because unhealthy lifestyles^4,5^. Therefore, the prevention and treatment of HLP warrants considerable attention.

Lipid regulators were once considered the gold standard for dyslipidemia^6,7^. Notably, statins, inhibitors of 3-hydroxy-3-methylglutaryl coenzyme A reductase (HMG-CoA), are effective in eliminating LDL-C from the blood^8,9^. However, the long-term use of statins has various side effects, such as cataracts, diabetes, muscle problems, and drug tolerance^10,11^. Therefore, alternative treatments for HLP are urgently needed.

**Figure 1:**
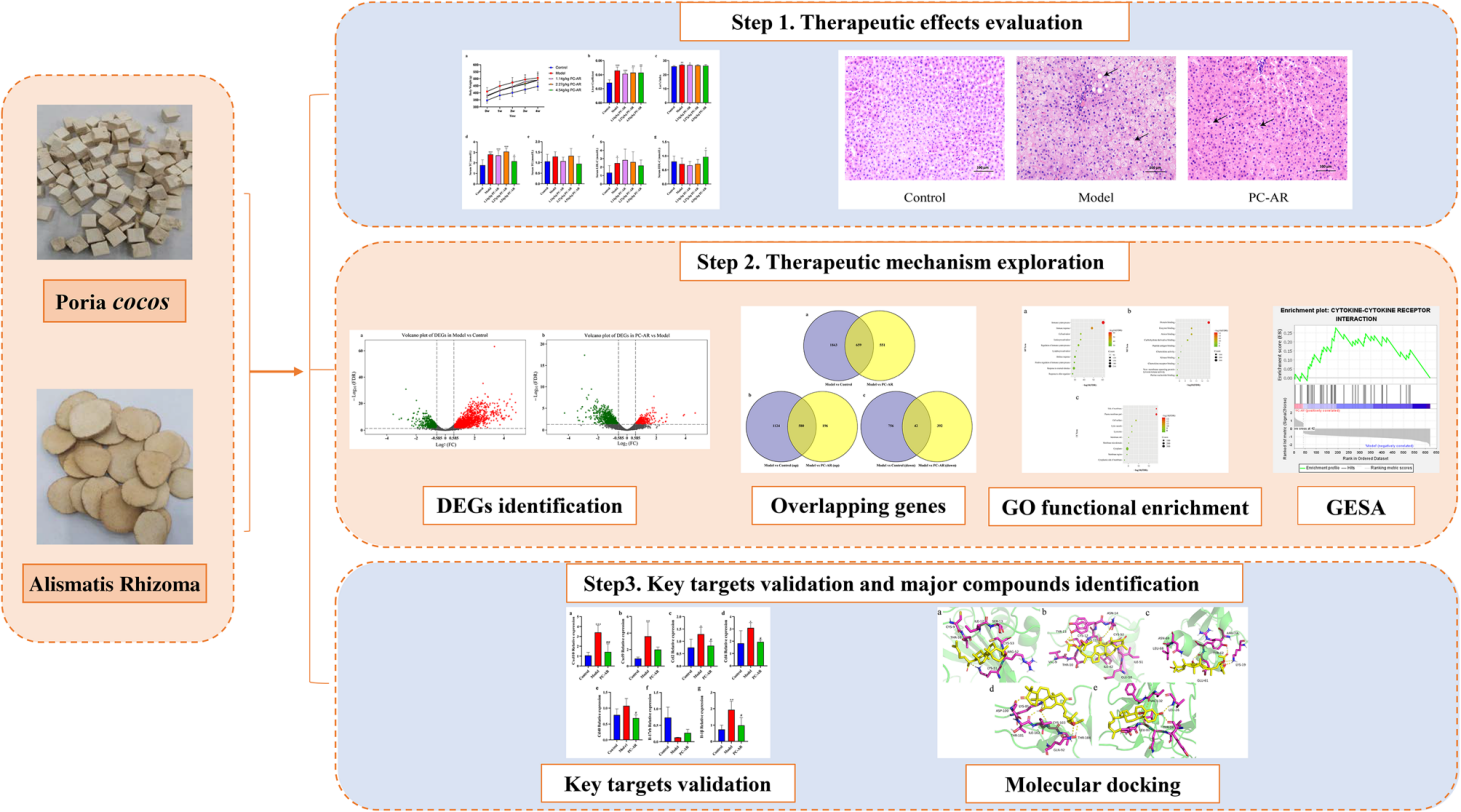
Schematic diagram of strategies for clarifying the therapeutic mechanism of PC and AR used as a combined treatment for HLP. PC, *Poria cocos*; AR, *Alismatis rhizoma*; HLP, hyperlipidemia.

Some Chinese medicines have well-established lipid-lowering effects^12^. *Poria cocos* (PC) is the dried sclerotia of the fungus *Poria cocos* (Schw.) Wolf, and *Alismatis Rhizoma* (AR) is the dried rhizome of *Alisma plantago-aquatica subsp. orientale* (Sam.) Sam^13^. These herbs were first recorded in *Shennong’s Classic of Materia Medica* and have been used for over 2000 years. According to Traditional Chinese Medicine (TCM) theory, the combination of PC and AR has the effect of invigorating the spleen and eliminating excessive moisture. PC and AR are commonly used collaboratively in prescriptions such as Wuling powder, Fuling Zexie decoction, and Liuwei Dihuang pills^14^. Wuling powder effectively reduced the weight and degree of obesity in patients with HLP^15^. Fuling Zexie decoction and Liuwei Dihuang pills improve serum lipid levels and protect the vascular endothelium^16–18^. In a complex network analysis of 453 medical cases, PC and AR were found to be key TCM pairs used to treat wet syndrome^19^. Studies on the active components of PC and AR have provided a basis for their combined use. Pachymic acid, pachymaran and other compounds extracted from PC have lipid-lowering, liver-protective, and anti-inflammatory effects^20–22^. Li et al. reported that 9 of 87 triterpenes with a high mean impact value had a significant lipid-lowering effect^23^. The main terpenoid monomers extracted from AR, 24-acetazenol A, 23-acetazenol C, and epoxisenol have anti-lipidemic effects on NAPCD cells^24^. At present, the use of these two herbal medicines individually is uncommon. To further investigate the synergistic therapeutic effects and underlying mechanism of the herbal pair PC–AR, this study integrated animal experiments, liver transcriptome technology, network-based analysis and molecular docking to identify potential disease biomarkers and candidate targets for HLP therapy (Fig. 1).

## Materials and methods

### Animals

Fifty 6-week-old male Sprague–Dawley (SD) rats were obtained from the Experimental Animal Science and Technology Development Co., Ltd., affiliated with Southern Medical University (license number: SCXK 2011-0015). The rats were housed in an SPF-grade barrier environment at an ambient temperature of 21–25℃ with 50–60% humidity and maintained on a 12-h light/12-h dark cycle in the Laboratory Animal Center of Guangzhou University of Chinese Medicine (Guangzhou, China). All animals protocols followed the Regulations of Experimental Animal Ethics Committee of Guangzhou University of Chinese Medicine and were approved by the Animal Ethics Committee (ethical review number: 00157477). The entire research process including experimental procedures and data analysis, met the Essential 10 and Recommended Set of ARRIVE Guidelines 2.0 criteria^25^.

### Equipment

The following equipment was used: a multifunctional microplate reader (Enspire 2300, PerkinElmer, MA, USA), paraffin embedding machine (Histocore Arcadiac, Leica, Wetzlar, Germany), automatic dehydrator (Excelsior AS, Thermo Fisher Scientific, MA, USA), paraffin slicing machine (RM 2245, Leica, Wetzlar, Germany); optical microscope (BX46F, Olympus, Tokyo, Japan), vertical electrophoresis system (Mini-Protean Tetra, Bio-Rad, CA, USA), and automatic chemiluminescence imaging system (Tanon 4600, Tanon, Shanghai, China).

### Construction of the HLP rat model

After one week of acclimatization, 40 rats were fed a high-fat diet (HFD) for four weeks. The HFD consisted of 52.2% maintenance feed, 0.4% premixed feed, 20% sucrose, 15% lard, 1.2% cholesterol, 0.2% sodium cholate, 10% casein, 0.6% dicalcium phosphate, and 0.4% mountain flour. The rats in control group (n = 10) were fed a chow diet. At the end of the fourth week, after the rats had fasted for 12 h, blood lipids indices, including TC, TG, LDL-C, and HDL-C, were analyzed to evaluate whether the model was successfully established. The model evaluation criteria were assessed as described previously^26^.

### Medicine preparation

PC (batch number: 190301) and AR (batch number: 190701), were purchased from Zisun Chinese Pharmaceutical Co. Ltd. (Guangzhou, China). The Inspection Certificate Reports of PC and AR (details in the Supplementary Material 1) indicated that herbs’ traits, identification, check items, extracts, and content met the standards of *Chinese Pharmacopoeia* (2015) section I–IV. A 15-g PC and 10-g AR mixture was pre-soaked in distilled water for 20 min and then extracted twice with ddH_2_O (1:10 and 1:8, w/v), producing a 0.454 g/mL crude drug extract. The extract was then diluted to 0.114 g/mL and 0.227 g/mL. All herbal solutions were stored at 4℃ until use.

The computational formula for determining the rat equivalent dose, according to body surface area based dosing^27^ (0.018, rat/human), is as follows:

1 $$Rat\, dose=\frac{human\, equivalent\, dose \times\, human\, body\, weight \times 0.018}{rat\, body\, weight}$$

The daily dose of PC–AR (25 g) for a person (70 kg) is 0.36 g/kg, which is equivalent to the 2.27 g/kg concentration used as the median dose for the rats (0.2 kg). The low and high doses of PC–AR were 1.14 g/kg and 4.54 g/kg respectively. The volume of the intragastric liquid administered daily to the rats was 1mL/100g.

### PC–AR treatment

Before the intervention was implemented, 50 male SD rats were divided into five groups (10 rats per group): control, model, 1.14g/kg PC–AR, 2.27g/kg PC–AR and 4.54g/kg PC–AR groups. In the control and model groups, PC–AR was replaced with an equal volume of 0.9% sterile saline. After four weeks of treatment, all rats were sacrificed, and blood and livers were harvested for further analysis.

### Phenotypic data and samples collection

The rats from all five groups were fasted overnight at the end of the PC–AR treatment period. The body weight (BW) of all rats was measured before anesthetization. The animals were anesthetized by an intraperitoneal injection (50 mg/kg) of 2% pentobarbital sodium (Guangzhou Qiyun Biological Technology Co. Ltd., No.GH-40C0). Body length measurement and blood samples collection were performed under general anesthesia. Thereafter, all the animals were euthanized for liver sample collection. After standing for 1–2 h, the collected blood was centrifuged at 4 ℃ and 3,500 rpm for 10 min. The upper serum was collected and stored in a refrigerator at 20 ℃. The excised livers were washed with cold normal saline, frozen in liquid nitrogen, and stored at −80˚C until use. The liver coefficient^28^ and Lee’s index^28^ were calculated after the experiment the following formulae:

2 $$\mathrm{Liver\, coefficient}=\frac{\mathrm{Liver\, weight }(\mathrm{g})}{\mathrm{Body\, weight }(\mathrm{g})}$$

3 $${\text{Lee}}\prime {\text{s}}\;{\text{index}} = \sqrt[3]{{\frac{{{\text{Body}}\;{\text{weight}} \left( {\text{g}} \right)*1000}}{{{\text{Body}}\;{\text{length}} \left( {{\text{cm}}} \right)}}}}$$

### Blood lipid measurement

TC, TG, LDL-C, and HDL-C concentrations were determined at the end of the fourth and eighth weeks using blood lipid detection kits provided by the Nanjing Jiancheng Biotechnology Company. Blood lipid detection was performed according to the manufacturer’s instructions.

### Hematoxylin and eosin (H&E) staining

Collected rat liver tissues were fixed in 4% paraformaldehyde for 24 h, dehydrated in gradient grade ethanol, cleared with xylene, embedded in paraffin, and cut into 4μm sections. The tissue slides were then dehydrated with xylene and gradient grade ethanol, incubated with hematoxylin for 5 min, stained with an eosin solution for 1–3 min, dehydrated again with gradient grade ethanol and xylene, and sealed with neutral gum. The liver cell nuclei (stained blue) and cytoplasm (stained red) were observed using a microscope.

### Transcriptome sequencing analysis

Total RNA was extracted from the livers using TRIzol solution (Thermo Fisher Scientific) following the manufacturer’s protocol. RNA purity was assessed by determining the absorbance at 260 and 280 nm using a spectrophotometer (IMPLEN, CA, USA). The RNA concentration was measured using the Qubit RNA Assay Kit along with the Qubit 2.0 Fluorometer (Life Technologies, Carlsbad, CA, USA), and RNA integrity was assessed using the Agilent Bioanalyzer 2100 system (Agilent Technologies). A cDNA library was constructed using PCR amplification. The quality of the cDNA library was confirmed by quantitation, using a Qubit 2.0. The insert size was detected using Agilent 2100 istrument, and the library concentration was confirmed by quantitative PCR. All the samples were sequenced on an Illumina HiSeq 2500 platform. Raw data with a Q30 base percentage of over 86.09% were obtained. The obtained clean reads were blasted and mapped against the reference genome database (Species: Rattus norvegicus; Version: Rnor_6.0) using the TopHat2^29^ software. The mapping rates of the different samples against the reference database were between 73.76% and 83.81%. Fragments per kilobase per million fragments mapped values were calculated and used to normalize gene expression levels.

### Screening of differentially expressed genes (DEGs)

The R studio software (v 1.1.463)^30^ was used for data processing. All gene expression values were transformed into fold change (FC for group comparisons, using the DESeq2 package (v 1.26.0)^31^. DEGs were defined according to the criteria |log2(FC)|> 0.585 and false discovery rate (FDR) 0.05. A volcano plot, processed using the ggplot2 package (v 3.4.1)^32^ was used to display the DEGs in both comparison sets based on the relationship between log10(FDR) and log2(FC). In addition, hierarchical clustering analysis was performed using the pheatmap package (v 1.0.12)^33^ to compare the DEGs among the groups. Raw sequencing data and processed data were uploaded to the Gene Expression Omnibus database (https://www.ncbi.nlm.nih.gov/geo/) under accession number GSE212771.

### Functional enrichment analysis

Gene ontology (GO) enrichment analysis was performed to determine the main mechanisms involved in the occurrence and treatment of HLP. First, all DEGs were mapped to GO terms in the GO database (http://www.geneontology.org/), falling under three categories, molecular function (MF), cellular component (CC), and biological process (BP). Gene numbers were calculated for every term, and significantly enriched GO terms were defined using a hypergeometric test. The calculated *P*-value were subjected to false discovery rate (FDR) correction, with Q ≤ 0.05 used, as a threshold. GO terms that met this criterion were defined as significantly enriched.

### Gene set enrichment analysis (GSEA)

GSEA was performed to verify which biologically significant gene sets were significantly associated with the different groups of samples. The annotated gene set for *rattus norvergicus* was acquired from the Kyoto Encyclopedia of Genes and Genomes (KEGG)^34^ database and processed as a GMT file using R software (v 4.0.2)^30^. Other files containing the gene expression data in .txt format and the phenotype labels in .cls format for the model vs. control and the model vs. PC–AR groups were loaded into the GSEA (v 4.3.2)^35^. Finally, following standard protocols, 1,000 permutations were performed for each analysis to identify enriched pathways based on gene expression.

### Real-time quantitative PCR (RT-qPCR) analysis

The RT-qPCR was used to detect the expressional levels of candidate targets. Total RNA was extracted from rat liver tissues using a lysis buffer with a Tissue RNA Purification Kit (#RN001A, EZBioscience) according to the manufacturer’s instructions. After determining the concentration and purity of the RNA using a nucleic acid quantitative analyzer (NanoDrop One, ThermoFisher Scientific Inc.), reverse transcription was conduceted using a Color Reverse Transcription Kit (#A0010CGQ, EZBioscience). RT-qPCR was then performed with (ROX2 Plus) 2xColor SYBR Green qPCR Master Mix (#A0012R2, EZBioscience) on an fluorescence quantitation PCR system (CFX96, Bio-Rad). Relative mRNA expression levels were normalized to β-actin (ACTB). The measurement results were expressed as 2^−△△CT^. Primer sequences used in this study are listed in Supplementary Material 2.

### Molecular docking

Compounds contained in PC and AR were collected from the Traditional Chinese Medicine Systems Pharmacology Database and Analysis Platform (TCMSP; http://www.tcmsp-e.com) ^36^according to oral bioavailability (OB) ≥ 30% and drug-like properties (DL) ≥ 0.18 criteria. Three-dimensional (3D) structure diagrams of the compounds were obtained from PubChem (https://pubchem.ncbi.nlm.nih.gov/). If no 3D structural diagram was available, two-dimensional (2D) structural diagram were used. The 2D structure diagram was converted to 3D using Chem3D software^37^. All structure diagrams were saved in PDB format. Next, the PDB structure files were imported into AutoDock4 (v 4.2.6)^38^ to add charging information and display rotatable keys. The files were saved in the required pdbqt format. The protein crystal structures corresponding to the core target genes were downloaded from the PDB database and imported into PyMOL^39^ (v 2.4.0 open-source; https://github.com/schrodinger/pymol-open-source) to remove water molecules and impurities, following the addition of hydrogen atoms in the AutoDock4 and pdbqt formats. These compounds were used as ligands and proteins as receptors for molecular docking analysis. AutoDock4 was used to estimate the binding capacities of the molecules and targets. The results were visualized using PyMOL software (v 2.4.0 open source).

### Statistical analysis

Statistical analysis was performed using SPSS statistical software (v22.0, SPSS, Inc, Chicago, IL, USA). All data were analyzed using one-way analysis of variance (ANOVA) after the normality test. If the data met the condition of homogeneity of variance condition, the least significant difference (LSD) and Tukey’s post-hoc tests were used for three and four comparison groups, respectively. Welch’s analysis of variance combined with the Games–Howell post hoc test was applied under conditions of heterogeneity of variance when there was a significant difference between the groups. Statistical data are presented as the mean ± standard deviation. A *P*-value of < 0.05 was defined as indicative of a significant difference.

### Declaration of third party material

Following the animal ethics principle of minimizing harm to animals, the control and model groups of rats used in this study were shared with the two groups previously described in published literature^26^. Permissions was obtained from the previous publisher. The medicine used in this study, *Poria cocos* and *Alismatis rhizoma*, differs from that used in the published literature. Additionally, the efficacy results, transcriptome data, comprehensive targets screening process and RT-qPCR validation results of PC–AR have not been published before, thus supporting for the originality of the study.

## Results

### Inhibition of high fat diet (HFD) -induced HLP progression by PC–AR

A rat model of HLP was successfully established after four weeks. Different doses (1.14 g/kg, 2.27 g/kg and 4.54 g/kg) of PC–AR were orally administered to the rats once per day for four weeks to determine the therapeutic effect of PC–AR on HFD-induced HLP. BW,the liver coefficient, Lee’s index, serum lipid levels and liver pathological morphology were compared after PC–AR treatment.

**Figure 2:**
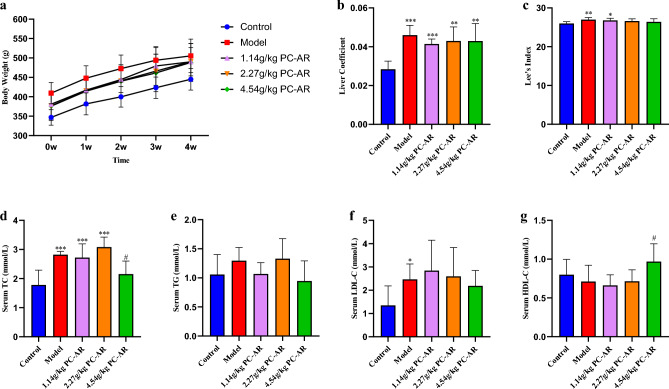
Changes in BW (**a**) in the control (n = 10), model (n = 10), 1.14 g/kg PC–AR (n = 10), 2.27 g/kg PC–AR (n = 10), and 4.54 g/kg PC–AR groups (n = 10) during the treatment period. After administration, liver coefficient (**b**) and Lee’s index (c), serum levels of TC (d), TG (e), LDL-C (f), and HDL-C (g) were determined. *P < 0.05, **P < 0.01, ***P < 0.001, compared with the control group; ^#^*P* < 0.05, compared with the model group. BW, body weight; TC, total cholesterol; TG, triglycerides; LDL-C, low-density lipoprotein cholesterol; HDL-C, high-density lipoprotein cholesterol.

There were no significant differences in BW changes among the four groups of HFD-fed rats before and after treatment (Fig. 2a). The liver coefficient (Fig. 2b) and Lee’s index (Fig. 2c) were lower in the 4.54 g/kg PC–AR group than in the model group, although the difference was not significant. Compared with the model group, the 4.54 g/kg PC–AR group exhibited a reversal in the accumulation of serum TC (*P* < 0.05) and an increase in the content of HDL-C (*P* < 0.05) (Fig. 2d-g). Only the 4.54 g/kg PC–AR treatment significantly affected serum lipid levels among the three interventions. Therefore, the 4.54 g/kg PC–AR dose group was selected for further research.

**Figure 3:**
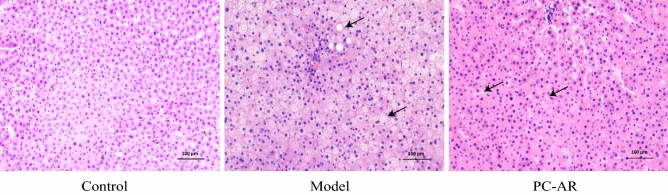
HE staining of liver tissues of rats in each group. The liver histology of rats in the different groups was compared to demonstrate the efficacy of PC–AR against HLP (200$\times$ magnification). The 4.54g/kg PC–AR group was selected as the PC–AR administration group. Compared with that in the control group, the degree of liver damage and hepatic steatosis was aggravated in the model group. In the PC–AR group, the pathological characteristics of the in liver cells were greatly alleviated, and a normal histology was recovered, compared to the model group. H&E, hematoxylin and eosin. Black arrows indicate the cytoplasmic vacuolation.

H&E staining (Fig. 3) was used to assess pathological changes in the liver. The morphology of liver cells and lobules in the control group was normal. The cells and lobules were distributed in an orderly manner, with no obvious lipid deposition. In the model group, the liver lobule structure was disordered. Lipid droplet vacuoles and lipid accumulation were common, with ballooning degeneration appearing in certain cells. Compared with that in the model group, the number of fat vacuoles and lipid deposits in the 4.54g/kg PC–AR group was significantly reduced ,indicating that the pathological morphology of hepatocytes induced by the HFD was significantly improved by PC–AR administration.

### Transcriptome analysis and screening of differentially expressed genes (DEGs)

**Figure 4:**
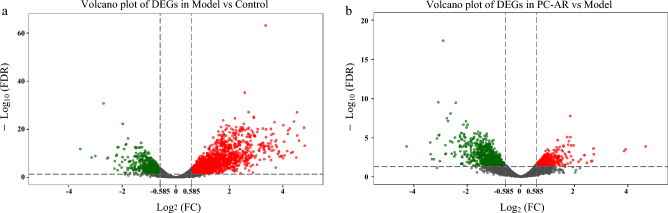
Volcano plots displaying DEGs based on the criteria of |log2(FC)|> 0.585 and FDR $<$ 0.05 in (**a**) model group versus control group (Model vs. Control)and (**b**) the PC–AR group versus model group (PC–AR vs. Model). Green spots indicate down-regulated genes, and red spots indicate up-regulated genes. FC, fold change; FDR, false discovery rate; DEG, differentially expressed gene.

Five control, five model, and six PC–AR samples, along with 11,436 genes, were included in this study. DEGs between different groups were identified, based on the criteria |log2(FC)|> 0.585 and FDR < 0.05 to demonstrate the molecular mechanisms of the effects of HLP and PC–AR, A total of 2502 HLP-related DEGs (1704 up-regulated and 798 down-regulated) were identified between the model and control groups (Fig. 4a and Supplementary Dataset S1). PC–AR—related DEGs (a total of 1210; 434 upregulated and 776 downregulated) were identified between the PC–AR and model groups (Fig. 4b and Supplementary ataset S2).

**Figure 5:**
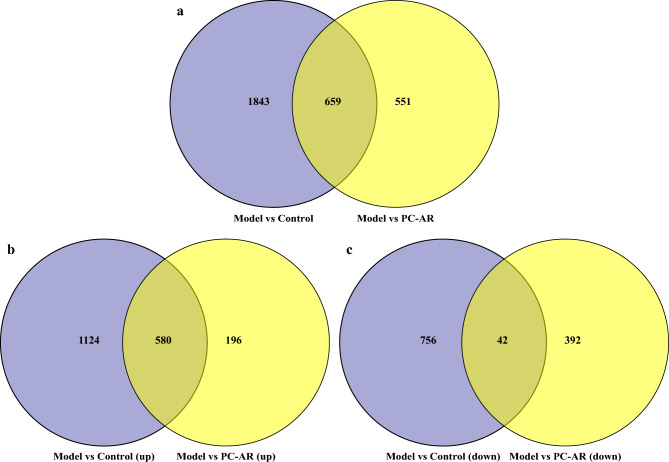
(**a**) The 659 overlapping DEGs between the model group versus the control group (Model vs. Control) and the model group versus PC–AR group (Model vs. PC–AR). (**b**) A total of 580 up-regulated DEGs were shared. (**c**) A total of 42 down-regulated DEGs were shared. PC–AR treatment effectively regulated 622 of the 659 DEGs.

Further analysis was conducted to compare the DEGs between the different groups. The model vs. control and PC–AR vs. model comparison sets shared 659 DEGs (Fig. 5a). To identify the genes that were effectively regulated by PC–AR, 622 genes that exhibited opposite trends in the model vs. control and PC–AR vs. model comparison sets were considered PC–AR regulated DEGs. The up-regulated genes were compared, and 580 overlapping DEGs were identified (Fig. 5b), indicating that PC–AR treatment reversed the expression of genes in the model group. Similarly, the down-regulated genes were compared, and 42 overlapping DEGs were identified (Fig. 5c). These 580 up-regulated DEGs and 42 down-regulated DEGs (622 in total), were termed “regulated genes”. These 622 genes and their corresponding gene symbols are listed in Supplementary Dataset S3. Hub genes associated with HLP and the effects of PC–AR on HLP progression in rats were identified. In addition, a heatmap (Supplementary Material 3) was plotted to illustrate the hierarchical clustering of genes and to compare the expression of the 622 DEGs among the three treatment groups. Genes that were highly expressed in the model group exhibited low expression levels in the control and PC–AR groups.

### Functional enrichment analysis results

**Figure 6:**
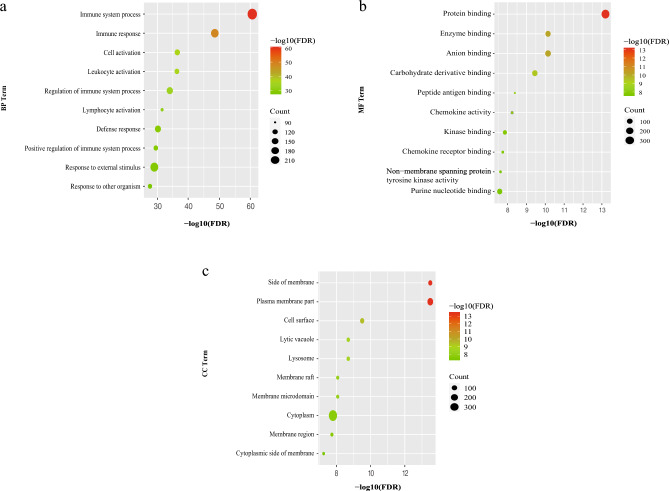
The top 10 ranked GO terms associated with the 622 regulated genes. (**a**) Top 10 BP terms; (**b**) top 10 MF terms; (**c**) top 10 CC terms. GO, gene ontology; BP, biological process; MF, molecular function; CC, cellular component, FDR or Q-value < 0.05.

The GO functional enrichment analysis of the 622 regulated genes was performed to further explore the mechanism underlying the PC–AR treatment’s effects on HLP,. The DEGs were significantly enriched in immune and inflammatory response-related annotations (*P* < 0.05). The top ten enriched BP terms (Fig. 6a) included the immune system and immune response. The MF terms (Fig. 6b) included protein and enzyme binding, and the enriched CC terms (Fig. 6c) included membrane and plasma membrane (the complete enrichment analysis data can be found in Supplementary Material 4).

### The enriched signaling pathway analyzed by GSEA

**Figure 7:**
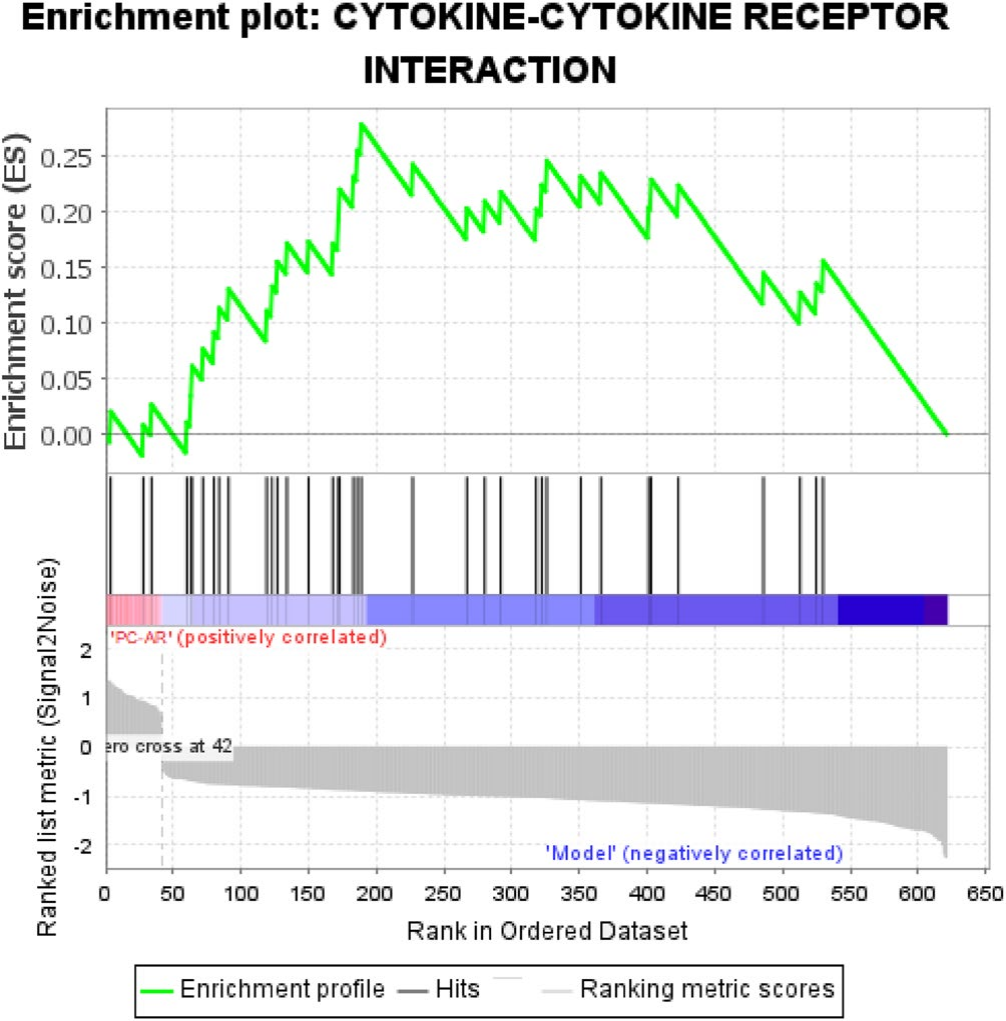
GSEA of DEGs in the PC–AR group versus the model group. GSEA, gene set enrichment analysis; KEGG, Kyoto Encyclopedia of Genes and Genomes.

GSEA was performed to show the enriched signaling pathway to confirm the pathway which may play a dominant role in the hyperlipidemic treatment of PC–AR,. The key KEGG pathway was identified by comparing the PC–AR and model groups regarding the expression of the 622 DEGs. Compared with the model group, the PC–AR group exhibited significant enrichment of the cytokine-cytokine receptor interaction pathway (Fig. 7 and Supplementary Material 5, NOM *P* < 0.05). The core symbols characterized by a “yes” that enriched in the cytokine-cytokine receptor interaction pathway are listed in Supplementary Material 6. In addition, the top five targets, Cxcl10, Ccl2, Cxcl9, Ccl4 and Cd40, significantly down-regulated by PC–AR according to their FC values (Table 1 and Supplementary Material 7) and were enriched in the cytokine-cytokine receptor interaction pathway, were identified as potential key targets for the validation of the anti-inflammation effects.

**Table 1:** Comparison of gene expression differences of five targets between the PC–AR group and model group.

Symbol	Fold change PC–AR vs model	FDR PC–AR vs model	Fold change control vs model	FDR control vs model	Regulation in two comparison parts
Cxcl10	0.095609	4.26E−05	0.035449	7.5813E−14	Down
Ccl2	0.215277	0.022889	0.044059	6.7314E−09	Down
Cxcl9	0.231895	0.001473	0.060381	1.7554E−15	Down
Ccl4	0.274342	0.013162	0.128018	8.7039E−09	Down
Cd40	0.299343	0.00296	0.187898	6.3126E−07	Down

### RT-qPCR analysis validated that PC–AR down-regulates the overexpression of cytokines

**Figure 8:**
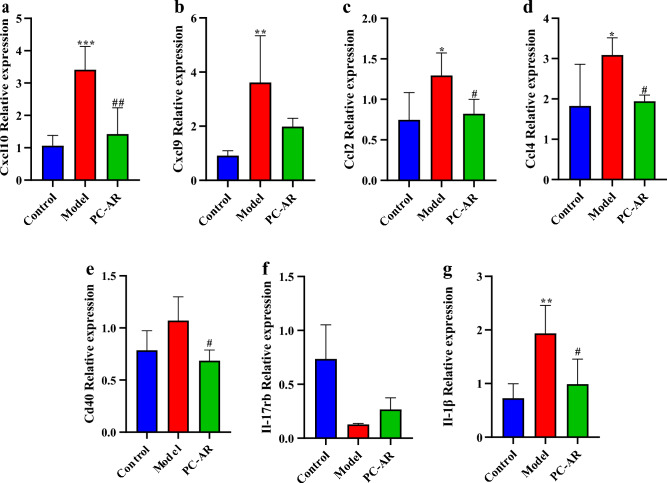
Relative expression of Cxcl10, Cxcl9, Ccl2, Ccl4, Cd40, Il-17rb and Il-1β mRNA (n = 4). *P < 0.05, **P < 0.01, ***P < 0.001, compared with the control group; ^#^*P* < 0.05, ^##^*P* < 0.01, compared with the model group.

Combined RNA-sequencing and GESA analyses identified the cytokine-cytokine receptor interaction pathway as the key pathway in the hyperlipidemia treatment of PC–AR. HFD causes the chronic injury and secretion of inflammatory cytokines with over-expression of Cxcl10, Cxcl9,Ccl2, Ccl4, and Cd40 (Fig. 8). C-C and C-X-C are the major subfamilies of chemokines that originate from the metabolically-injured hepatocytes and contribute to liver inflammation^40^. As shown in Fig. 8, PC–AR significantly decreased the expression of Cxcl10, Ccl2, and Ccl4 (*P* < 0.05) and partly exerted the inhibition effect on the abnormal secretion of Cxcl9. Besides, PC–AR reversed the expression of Cd40 and Il-1β mRNA (*P* < 0.05), and slightly upregulated the Il-17rb mRNA, further indicating the inhibitory effect on liver inflammation.

### Molecular docking analysis

Molecular docking analysis was applied to further explore the major compounds of PC–AR that play a role in its therapeutic effect on hyperlipidemia in rats and the potential binding patterns of the major compounds and key targets. A total of 13 compounds meeting the criteria of OB ≥ 30% and DL ≥ 0.18 were identified as major active compounds (Supplementary Material 8) , binding to the Cxcl10, Ccl2, Ccl4, Cd40 and Il-1β key targets (Supplementary Material 9).

**Figure 9:**
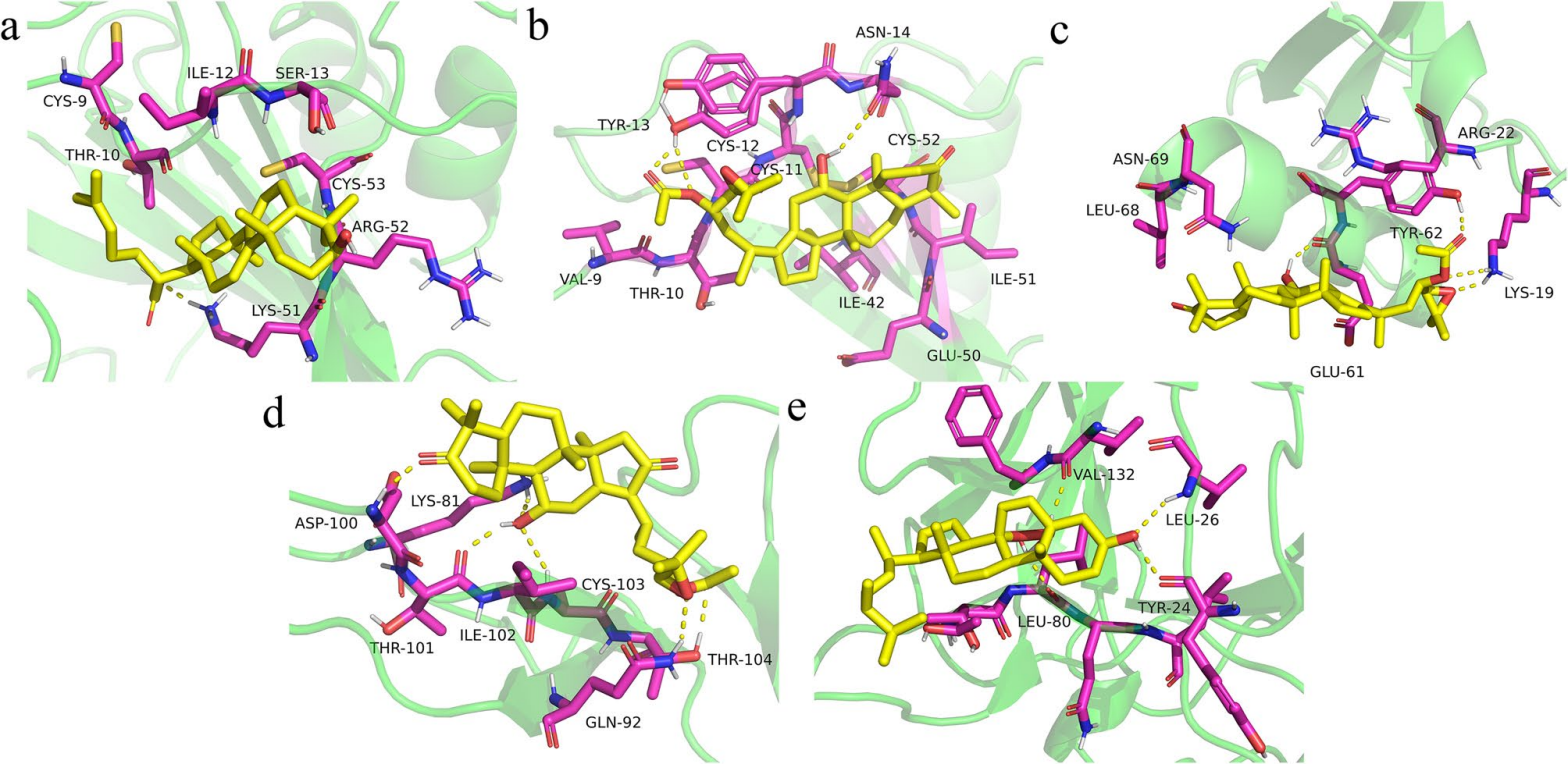
Schematic 3D representation of molecular docking models: (**a**) Trametenolic acid (Pubchem Cid: 125181708) to Cxcl10 (PDB ID: 1O7Y), (**b**) Alisol B 23-acetate (Pubchem Cid: 14036811) to Ccl2 (PDB id:1DOK), (**c**) Alisol B monoacetate (Pubchem Cid: 163083573) to Ccl4 (PDB ID: 2X6L), (**d**) Alisol C monoacetate (Pubchem Cid: 14036813) to Cd40 (PDB ID: 7P3I), (**e**) Ergosterol peroxide (Pubchem Cid: 5351516) to Il-1β (PDB ID: 5R8Q). The molecular docking results were visualized using PyMOL software (v 2.4.0 open source; https://github.com/schrodinger/pymol-open-source).

A schematic of the 3D representation of the molecular docking model with the lowest binding energy for each target to which the compound binds is shown in Fig. 9. The energies for binding trametenolic acid to Cxcl10 was -7.15 kcal/mol (Fig. 9a), for Alisol B 23-acetate to Ccl2 was -7.98 kcal/mol (Fig. 9b), for alisol B monoacetate to Ccl4 was -6.83 kcal/mol (Fig. 9c), for alisol C monoacetate to Cd40 was -7.29 kcal/mol (Fig. 9d) and for ergosterol peroxide to Il-1β was -8.49 kcal/mol (Fig. 9e).

## Discussion

The HFD-induced HLP model used in this study was established using our previously developed modeling method^41,42^. This HLP model was the most consistent with the clinical characteristics of human HLP^43^. At the end of the modeling period, compared with those in the control group, the levels of TC and TG in the model group increased, and the HDL level decreased (*P* < 0.01), based on the criteria for establishing a mixed HLP animal model published in the “Evaluation Method of Auxiliary Hypolipidemic Function”^44^. After four weeks of high-dose PC–AR administration, serum TC level was reduced, and HDL-C level was significantly elevated (*P* < 0.05). Lee’s index and liver morphology also indicated the therapeutic efficacy of PC–AR. However, the dose–effect relationship was not clearly defined, and further research using more samples is required. To explore the targets and mechanism of action of PC–AR, transcriptomic analysis was performed. PC–AR improved the phenotypic characteristics of rats with HLP and altered gene expression. A total of 622 DEGs were identified as effectively regulated genes; these genes exhibited opposite expression patterns in the model group, compared to those in the other two groups.

Gene annotation and functional analysis of the 622 overlapping genes were performed to better understand the mechanism underlying the effect of PC–AR on HLP. GSEA of the 622 DEGs strongly indicated that PC–AR treatment of HLP in rats may function, in part, by regulating the cytokine-cytokine receptor interaction pathway, which is closely related to the inflammatory response. The core targets enriched in this pathway (Supplementary Material 6) included chemokines (Cxcl10, Cxcl9, Ccl2, Ccl4, Ccl3 and others), chemokine receptors (Ccr7 and Ccr5), cytokine receptors (Il17rb, Il3ra, Il15ra) and tumor necrosis factor ligand superfamily (Tnfsf10 and Tnfsf13). It is best known for the ability of chemokines to induce cell migration and is generally thought to be involved in all protective or destructive immune and inflammatory responses^45^. The enrichment results indicate a close relationship between dyslipidemia and inflammatory disorders, providing a clue for PC–AR achieving a inflammatory response regulation simultaneously with dyslipidemia intervention.

When an overabundance of free fatty acids impairs the storage capacity of adipose tissue, it leads to excess deposition of free fatty acids in the form of triglycerides in non-adipose tissue, resulting in lipotoxic damage to the tissue. Lipotoxic damage occurs in hepatocytes, priming immune cells, and inducing cytokines and chemokines release, ultimately leading to an immune cascade^46^. Kupffer cells are liver-resident macrophages that are responsible for maintaining liver homeostasis, clearing pathogens and regulating iron metabolism^47^. As one of the phenotypes of macrophage polarization, M1 macrophages are generally known as pro-inflammatory macrophages, secreting the pro-inflammatory cytokines, such as IL-1β, CCL2 ~ 5, CXCL8 ~ 10 and TNF-α^48^. The relationship between lipid metabolism and inflammatory responses in the liver may be related to liver damage induced by toxic fatty acids and the activation of Kupffer cells through damage-associated molecular patterns (DAMPs) that interact with toll-like receptors^49^. In addition, the synthesis of these cytokines is regulated by the ligation of CD40 on monocytes and macrophages which upregulates the production of matrix metalloproteinases (MMPs) and nitric oxide (NO)^50^. IL-17RB is the receptor of IL-25, a member of the IL-17 cytokine family, which constitutes the IL-25 signaling that develops beige adipose tissue. It was reported that the administration of IL-25 may increase beige fat and elevate adipose tissue thermogenesis in the way of alternately activating macrophages (from M1 to M2 polarization)^51^. In the present investigation, the expression of Cxcl10, Cxcl9, Ccl2, Ccl4, Il-1β and Cd40 mRNA increased and Il-17rb mRNA decreased in HFD-fed rats, suggesting that long-term HFD feeding induces an inflammatory response in the liver that gives rise to the production of pro-inflammatory cytokines. Our findings revealed that PC–AR administration reduced the expression of pro-inflammatory cytokines. This indicates the herbal pair PC–AR exerted the immunemodulatory effect on the liver response induced by high-fat diet exposure. At the same time, these results also suggest that PC–AR may affect the polarization of macrophages, which needs further investigation in the future.

Accumulating studies have elucidated that *Poria cocos* possess anti-inflammatory, immunomodulatory, anticancer and antihyperglycemic pharmacological properties^52^. In this study, the binding energy of trametenolic acid (one of Lanostane-type triterpenes) with Cxcl10 was −7.15 kcal/mol; the docking results with the other four targets are less than −5.0 kcal/mol, showing that this active compound has a good affinity with these targets. *Alismatis rhizome,* which contain more than 200 bioactive components (terpenoids in particular), have anti-inflammatory and antioxidant properties^53^. Aliso B 23-acetate and alisol C monoacetate, the active compounds extracted from *Alismatis rhizome*, were predicted to have a good affinity with Ccl2 and Cd40. Other components interacted well with these regulated targets (Supplementary Material 9). As shown in the current study, the treatment of HLP by PC–AR works through the effects of multiple components on multiple targets, the docking results provide predictive function for the subsequent selection of major core active ingredients. Previous studies have shown that the monomeric components extracted from isolated Poria cocos or Alismatis rhizome have the the function of regulating blood lipids^54,55^, and the current results after combination use suggest that PC–AR is associated with improvements in blood lipids, liver damage, and cytokine regulation, it is regrettable that a direct comparison between monotherapy or monomers and combined ones has not yet been made to prove the synergistic effects.

There are some limitations to our study. First, the active compounds of PC or AR are collected from databases and did not reflect all components of PC–AR, and future studies should apply advanced technologies to identify the certain components of PC and AR when mixed for the water extarction. Further, the water extraction methods should be compared with other methods to identify the extraction efficiency of the water extraction. Lastly, more comparison groups need to be set up in the future to compare the difference between Poria cocos or Alismatis rhizome alone and the combination of PC–AR.

## Conclusions

The improved serum lipid levels, Lee’s index, and liver pathological histology of rats in the PC–AR group indicate that PC–AR is a promising candidate for treating HLP. In addition, bioinformatics analysis of DEGs in rat livers suggested the potential of PC–AR to suppress HFD-induced immune and inflammatory responses. Cxcl10, Ccl2, Ccl4, Cd40 and Il-1β may be key targets of PC–AR in treating HLP.

### Supplementary Information

Supplementary Information 1.

Supplementary Information 2.

## Data Availability

The microarray datasets used in the current study can be searched out from https://www.ncbi.nlm.nih.gov/, the accession ID: GSE212771. The processed files are presented in supplemental tables. Contact the corresponding author for permission and quote the articles for the reasonable usage of the datasets.

## Abbreviations

BP: Biological process
CC: Cellular component
DEGs: Differentially expressed genes
FC: Fold change
FDR: False discovery rate
PC–AR: Herbal pair *Poria cocos (PC)* and *Alismatis Rhizoma (AR)*
FPKM: Fragments per kilobase per million fragments mapped
GEO: Gene Expression Omnibus
GO: Gene ontology
GSEA: Gene set enrichment analysis
HDL-C: High-density lipoprotein cholesterol
H&E: Hematoxylin and eosin staining
HFD: High-fat diet
HLP: Hyperlipidemia
HMG-CoA: 3-Hydroxy-3-methylglutaryl coenzyme A reductase
KEGG: Kyoto Encyclopedia of Genes and Genomes
LDL-C: Low-density lipoprotein cholesterol
Model vs Control: Model group versus control group
Model vs PC–AR: Model group versus PC–AR group
MF: Molecular function
PPI: Protein–protein interaction
SD: Sprague–Dawley
TC: Total cholesterol
TCM: Traditional Chinese medicine
TG: Triglycerides
PCR: Real-time quantitative PCR
