# Supplementary material for: Efficacy of Poria cocos and Alismatis rhizoma against diet-induced hyperlipidemia in rats based on transcriptome sequencing analysis
Source: Sci Rep. 2023 Oct 15;13:17493. doi: 10.1038/s41598-023-43954-6 (PMC10577139; doi:10.1038/s41598-023-43954-6)
Supplement: Supplementary file 2 — Supplementary Information 2. [file 41598_2023_43954_MOESM2_ESM.pdf]

Supplementary Material 1. The Inspection Certificate Reports of Poria cocos and Alismatis Rhizoma.

Zisun Chinese Pharmaceutical Co., Ltd. (Guangzhou, China)

Inspection Report

File Number: R-QM-045-02

Report number: C171221010

Inspection product name	Poria cocos (PC)	Inspection number	C171202
Place of origin	Anhui	Batch number	171202
Sample supply institusion	Zisun Chinese Pharmaceutical Co., Ltd. (Guangzhou, China)	Specification	/
Inspection purpose	Normal inspection	Packaging Materials	Composite packaging bag
Inspection item	Full inspection	Inspection date	2017/12/20
Inspection basis	***Chinese Pharmacopoeia*** (2015) section I-IV		
Inspection item	Standard regulation	Inspect result	
【Traits】	It should be characterized with the appearance of Poria (Poria piece)	Compliance	
【Identification】			
(1) Microscopic identification	It should have the microscopic characteristics of Poria	Compliance	
(2) Physical and chemical identification	It should meet the regulations	Compliance	
(3) Thin layer identification	Poria cocos should be detected	Compliance	
【Check Items】			
(1) Moisture content	No more than 18.0%	14.3%	
(2) Total ash	No more than 2.0%	0.2%	
(3) Sulfur dioxide residues	No more than 150mg/kg	42mg/kg	
【Extract】	Calculated as filtrated product, not less than 2.5%	3.1%	
Remark	Among these above items, the results of【Identification】【Check Item】(except moisture content) and【Extract】cited from the inspect results of the product to be packaged. The report number of product to be packaged is Y170920015.		
Inspection conclusion	This product is tested according to ***Chinese Pharmacopoeia*** (2015) section I-IV, and the results meet the regulations.		
Authorized signatory		Date of issue	2017/12/21

Zisun Chinese Pharmaceutical Co., Ltd. (Guangzhou, China)

Inspection Report

File Number：R-QM-045-02

Report number：C171213010

Inspection product name	Alismatis Rhizoma (AR)	Inspection number	C171201
Place of origin	Jiangxi	Batch number	171201
Sample supply institution	Zisun Chinese Pharmaceutical Co., Ltd. (Guangzhou, China)	Specification	/
Inspection purpose	Normal inspection	Packaging Materials	Composite packaging bag
Inspection item	Full inspection	Inspection date	2017/12/8
Inspection basis	***Chinese Pharmacopoeia*** (2015) section I-IV		
Inspection item	Standard regulation	Inspect result	
【Traits】	It should be characterized with the appearance of Alismatis Rhizoma.	Compliance	
【Identification】			
Microscopic identification	It should have the microscopic characteristics of Alismatis Rhizoma.	Compliance	
Thin layer identification	23-acetyl alisatol B should be detected.	Compliance	
【Check Item】			
(1) Moisture content	No more than 12.0%	10.1%	
(2) Total ash	No more than 5.0%	2.3%	
(3) Carbon dioxide residue	No more than 150mg/kg	7mg/kg	
【Extract】	Calculated as filtrated product, no less than 10.0%	10.7%	
【Content determination】			
Calculated on the anhydrous substance			
23-acetyl alisatol B	Not less than 0.050%	0.125%	
Remark	Among these above items，the results of【Identification】and【Check Item】(except moisture content) cited from the inspect results of the product to be packaged. The report number of product to be packaged is Y171106005.		
Inspection conclusion	This product is tested according to ***Chinese Pharmacopoeia*** (2015) section I-IV, and the results meet the regulations.		
Authorized signatory		Date of issue	2017/12/13

Supplementary Material 2. Sprague-Dawley rat specific primers used in this study**.**

gene	Forward primer（5'-3'）	Reverse primer（5'-3'）
ACTB	TGTCACCAACTGGGACGATA	GGGGTGTTGAAGGTCTCAAA
Ccl2	CTCACCTGCTGCTACTCATTCACTG	CTTCTTTGGGACACCTGCTGCTG
Ccl4	CTCCCACTTCCTGCTGCTTCTC	AGGCTGCTGGTCTCATAGTAATCC
Cd40	TGGCTGCTCTGATCTCGCTCTG	CCTAGATGGACCGCTGTCAACAAG
Cxcl10	TGAAAGCGGTGAGCCAAAGAAGG	CTGGGTAAAGGGAGGTGGAGAGAC
Cxcl9	AGTCCGTTGCTCTATTCCTCATGG	TGCCTTGGCTGGTGCTGATG
Il-17rb	CTGGTGCTGCTGGTGGCTAC	GATGTCTTTGTGCTCCTTCCTTGC
Il-1β	TCTCACAGCAGCATCTCGACAAG	CCACGGGCAAGACATAGGTAGC

Supplementary Material 3. Heatmap analysis of 622 regulated DEGs in three groups.

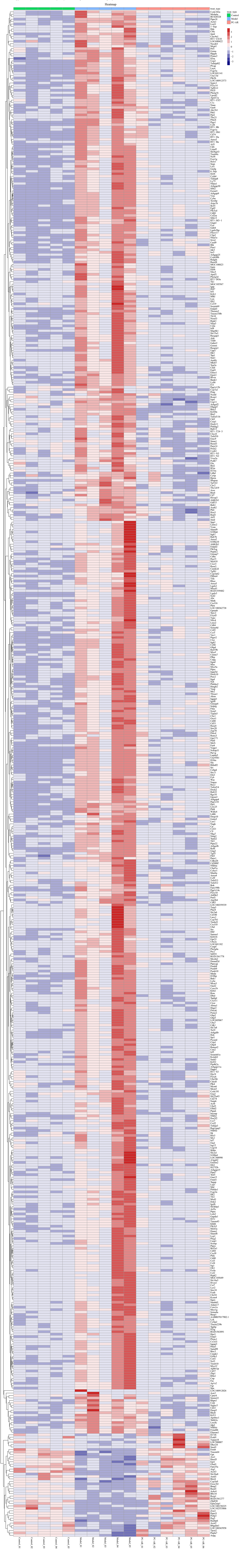

Supplementary Material 4. Top 10 annotation terms of Gene Ontology (GO) results of 622 DEGs

ID	Term	Count	FDR/*Q*-value
**Biological process**			
GO:0002376	immune system process	219	2.22E-61
GO:0006955	immune response	159	3.40E-49
GO:0001775	cell activation	112	2.72E-37
GO:0045321	leukocyte activation	105	3.71E-37
GO:0002682	regulation of immune system process	128	6.70E-35
GO:0046649	lymphocyte activation	90	2.98E-32
GO:0006952	defense response	125	6.62E-31
GO:0002684	positive regulation of immune system process	103	3.26E-30
GO:0009605	response to external stimulus	169	9.84E-30
GO:0051707	response to other organism	100	2.49E-28
**Molecular function**			
GO:0005515	protein binding	343	6.41E-14
GO:0019899	enzyme binding	116	7.17E-11
GO:0043168	anion binding	143	7.17E-11
GO:0097367	carbohydrate derivative binding	119	3.59E-10
GO:0042605	peptide antigen binding	12	4.08E-09
GO:0008009	chemokine activity	13	5.75E-09
GO:0019900	kinase binding	52	1.39E-08
GO:0042379	chemokine receptor binding	14	1.79E-08
GO:0004715	non-membrane spanning protein tyrosine kinase activity	13	2.38E-08
GO:0017076	purine nucleotide binding	103	2.60E-08
**Cellular component**			
GO:0098552	side of membrane	56	3.28E-14
GO:0044459	plasma membrane part	136	3.28E-14
GO:0009986	cell surface	61	3.12E-10
GO:0005764	lysosome	38	2.01E-09
GO:0000323	lytic vacuole	38	2.01E-09
GO:0045121	membrane raft	31	8.32E-09
GO:0098857	membrane microdomain	31	8.32E-09
GO:0005737	cytoplasm	349	1.58E-08
GO:0098589	membrane region	31	1.82E-08
GO:0098562	cytoplasmic side of membrane	22	5.56E-08

Supplementary Material 5. Gene set enrichment analysis results of 622 DEGs in the PC-AR versus model group, based on KEGG pathway annotation.

Name	Size	ES	NES	NOM P-val	FDR q-val	FWER P-val	Rank at max	Leading edge
**Enrichment in PC-AR group**								
CYTOKINE-CYTOKINE RECEPTOR INTERACTION	37	0.28	1.84	0.002	0.149	0.131	189	tags=57%, list=30%, signal=77%

Supplementary Material 6. Gene set enrichment analysis results of symbols in cytokine-cytokine receptor interaction pathway in the PC-AR group versus the model group.

NAME	SYMBOL	RANK IN GENE LIST	RANK METRIC SCORE	RUNNING ES	CORE ENRICHMENT
row_0	Bmp2	4	1.316683	0.020189	Yes
row_1	Ackr4	28	0.922368	0.0079	Yes
row_2	Il17rb	34	0.842879	0.02638	Yes
row_3	Ccl2	60	-0.65619	0.010672	Yes
row_4	Ccl3	63	-0.67769	0.03428	Yes
row_5	Cxcl16	64	-0.67805	0.061307	Yes
row_6	Ccl4	72	-0.7122	0.076369	Yes
row_7	Cxcl10	80	-0.73947	0.09143	Yes
row_8	Tnfsf10	84	-0.74396	0.113329	Yes
row_9	Il21r	91	-0.75193	0.130099	Yes
row_10	Cx3cl1	119	-0.79046	0.110973	Yes
row_11	Tnfsf13	123	-0.79621	0.132871	Yes
row_12	Tgfb1	127	-0.79729	0.15477	Yes
row_13	Ccr5	134	-0.80308	0.171541	Yes
row_14	Ccr7	150	-0.82488	0.172927	Yes
row_15	Cxcl9	168	-0.85461	0.170894	Yes
row_16	Il3ra	172	-0.85788	0.192793	Yes
row_17	Il15ra	173	-0.8603	0.21982	Yes
row_18	Ccl9	183	-0.86692	0.231462	Yes
row_19	Cd40	186	-0.87392	0.25507	Yes
row_20	Crlf2	189	-0.87756	0.278679	Yes
row_21	Il2rg	227	-0.92899	0.242458	No
row_22	Gdf15	267	-0.97162	0.202818	No
row_23	Cxcl11	280	-0.98171	0.209332	No
row_24	Il10ra	292	-0.99062	0.217556	No
row_25	Ccl6	318	-1.00969	0.201848	No
row_26	Csf2ra	322	-1.01153	0.223747	No
row_27	Cxcr3	326	-1.01669	0.245646	No
row_28	Tnfsf12	351	-1.05834	0.231647	No
row_29	Ifngr2	366	-1.07874	0.234742	No
row_30	Ccl19	401	-1.12117	0.20365	No
row_31	Il2rb	403	-1.12318	0.228967	No
row_32	Tnfrsf11b	423	-1.15148	0.223516	No
row_33	Fas	486	-1.25565	0.14456	No
row_34	Ebi3	513	-1.30385	0.127143	No
row_35	Ccl21	525	-1.32614	0.135366	No
row_36	Tnfrsf14	530	-1.33525	0.155556	No

Supplementary Material 7. Comparison of gene expression differences of all core enriched targets in the cytokine-cytokine receptor interaction pathway in two comparison parts.

Symbol	Fold change PC-AR vs model	FDR PC-AR vs model	Fold change PC-AR vs model	FDR control vs model	Regulation in two comparison parts
Cxcl10	0.095609	4.26E-05	0.035449	7.5813E-14	down
Ccl2	0.215277	0.022889	0.044059	6.7314E-09	down
Cxcl9	0.231895	0.001473	0.060381	1.7554E-15	down
Ccl4	0.274342	0.013162	0.128018	8.7039E-09	down
Cd40	0.299343	0.00296	0.187898	6.3126E-07	down
Tnfsf10	0.31768	0.010106	0.453679	0.00811309	down
Ccl3	0.32753	0.025148	0.069619	5.1127E-11	down
Cx3cl1	0.366583	0.01596	0.285768	8.0507E-05	down
Ccr7	0.383861	0.020353	0.315016	0.00076618	down
Il21r	0.415626	0.034418	0.205861	9.7619E-06	down
Cxcl16	0.459264	0.022543	0.160474	7.7378E-11	down
Il15ra	0.468581	0.023404	0.585345	0.03210601	down
Ccr5	0.476336	0.018323	0.240835	7.4818E-13	down
Tnfsf13	0.549231	0.031644	0.457221	0.00051531	down
Ccl9	0.550014	0.020912	0.56513	0.00049142	down
Tgfb1	0.613859	0.027143	0.340953	3.631E-13	down
Crlf2	0.618714	0.035904	0.50629	3.5692E-06	down
Il3ra	0.639109	0.032464	0.606415	0.00188604	down
Il17rb	3.303793	0.004133	2.190166	0.00107903	up
Ackr4	2.215139	0.009768	1.696584	0.01331381	up
Bmp2	2.061734	0.000498	1.896972	3.3249E-07	up

Supplementary Material 8. Major active compounds of Poria cocos and Alismatis Rhizoma meeting the criteria of OB ≥30% and DL≥ 0.18.

Herb	Mol ID	Component	MW	OB(%)	DL
Poria cocos	MOL000282	ergosta-7,22E-dien-3beta-ol	398.74	43.51	0.72
	MOL000283	Ergosterol peroxide	430.74	40.36	0.81
	MOL000275	trametenolic acid	456.78	38.71	0.80
	MOL000279	Cerevisterol	430.74	37.96	0.77
	MOL002464	1-Monolinolein	354.59	37.18	0.30
	MOL000296	hederagenin	414.79	36.91	0.75
Alismatis Rhizoma	MOL000359	sitosterol	414.79	36.91	0.75
	MOL000853	alisol B	444.72	36.76	0.82
	MOL000831	Alisol B monoacetate	514.82	35.58	0.81
	MOL000862	[(1S,3R)-1-[(2R)-3,3-dimethyloxiran-2-yl]-3-[(5R,8S,9S,10S,11S,14R)-11-hydroxy-4,4,8,10,14-pentamethyl-3-oxo-1,2,5,6,7,9,11,12,15,16-decahydrocyclopenta[a]phenanthren-17-yl]butyl] acetate	514.82	35.58	0.81
	MOL000856	alisol C monoacetate	514.77	33.06	0.83
	MOL000849	16β-methoxyalisol B monoacetate	544.85	32.43	0.77
	MOL000273	(2R)-2-[(3S,5R,10S,13R,14R,16R,17R)-3,16-dihydroxy-4,4,10,13,14-pentamethyl-2,3,5,6,12,15,16,17-octahydro-1H-cyclopenta[a]phenanthren-17-yl]-6-methylhept-5-enoic acid	470.76	30.93	0.81

Supplementary Material 9. Molecular docking analysis results of the major compounds and core targets.

Herb	Mol ID	Component	Pubchem Cid	Gene	PDB ID	binding energy (kcal/mol)
Alismatis Rhizoma	MOL002464	1-Monolinolein	6436630	Cxcl10	1O7Y	-4.79
Alismatis Rhizoma	MOL000856	Alisol C monoacetate	14036813	Cxcl10	1O7Y	-5.71
Poria cocos	MOL000282	Ergosta-7,22E-dien-3beta-ol	5283628	Cxcl10	1O7Y	-5.96
Alismatis Rhizoma	MOL000853	Alisol B	15558620	Cxcl10	1O7Y	-5.97
Alismatis Rhizoma	MOL000359	Sitosterol	12303645	Cxcl10	1O7Y	-6.07
Alismatis Rhizoma	MOL000862	[(1S,3R)-1-[(2R)-3,3-dimethyloxiran-2-yl]-3-[(5R,8S,9S,10S,11S,14R)-11-hydroxy-4,4,8,10,14-pentamethyl-3-oxo-1,2,5,6,7,9,11,12,15,16-decahydrocyclopenta[a]phenanthren-17-yl]butyl] acetate	14036811	Cxcl10	1O7Y	-6.18
Alismatis Rhizoma	MOL000849	16β-methoxyalisol B monoacetate	162953612	Cxcl10	1O7Y	-6.22
Poria cocos	MOL000283	Ergosterol peroxide	5351516	Cxcl10	1O7Y	-6.23
Poria cocos	MOL000279	Cerevisterol	10181133	Cxcl10	1O7Y	-6.4
Alismatis Rhizoma	MOL000831	Alisol B monoacetate	163083573	Cxcl10	1O7Y	-6.48
Poria cocos	MOL000273	(2R)-2-[(3S,5R,10S,13R,14R,16R,17R)-3,16-dihydroxy-4,4,10,13,14-pentamethyl-2,3,5,6,12,15,16,17-octahydro-1H-cyclopenta[a]phenanthren-17-yl]-6-methylhept-5-enoic acid	10743008	Cxcl10	1O7Y	-6.72
Poria cocos	MOL000296	Hederagenin	73299	Cxcl10	1O7Y	-6.76
Poria cocos	MOL000275	Trametenolic acid	125181708	Cxcl10	1O7Y	-7.15
Alismatis Rhizoma	MOL002464	1-Monolinolein	6436630	Ccl2	1DOK	-6.06
Poria cocos	MOL000296	Hederagenin	73299	Ccl2	1DOK	-6.83
Poria cocos	MOL000282	Ergosta-7,22E-dien-3beta-ol	5283628	Ccl2	1DOK	-6.98
Poria cocos	MOL000273	(2R)-2-[(3S,5R,10S,13R,14R,16R,17R)-3,16-dihydroxy-4,4,10,13,14-pentamethyl-2,3,5,6,12,15,16,17-octahydro-1H-cyclopenta[a]phenanthren-17-yl]-6-methylhept-5-enoic acid	10743008	Ccl2	1DOK	-7.21
Poria cocos	MOL000279	Cerevisterol	10181133	Ccl2	1DOK	-7.24

Alismatis Rhizoma	MOL000849	16β-methoxyalisol B monoacetate	162953612	Ccl2	1DOK	-7.59
Alismatis Rhizoma	MOL000853	Alisol B	15558620	Ccl2	1DOK	-7.66
Alismatis Rhizoma	MOL000856	Alisol C monoacetate	14036813	Ccl2	1DOK	-7.73
Poria cocos	MOL000275	Trametenolic acid	125181708	Ccl2	1DOK	-7.73
Alismatis Rhizoma	MOL000359	Sitosterol	12303645	Ccl2	1DOK	-7.76
Alismatis Rhizoma	MOL000831	Alisol B monoacetate	163083573	Ccl2	1DOK	-7.8
Poria cocos	MOL000283	Ergosterol peroxide	5351516	Ccl2	1DOK	-7.89
Alismatis Rhizoma	MOL000862	[(1S,3R)-1-[(2R)-3,3-dimethyloxiran-2-yl]-3-[(5R,8S,9S,10S,11S,14R)-11-hydroxy-4,4,8,10,14-pentamethyl-3-oxo-1,2,5,6,7,9,11,12,15,16-decahydrocyclopenta[a]phenanthren-17-yl]butyl] acetate	14036811	Ccl2	1DOK	-7.98
Alismatis Rhizoma	MOL002464	1-Monolinolein	6436630	Ccl4	2X6L	-4.59
Alismatis Rhizoma	MOL000853	Alisol B	15558620	Ccl4	2X6L	-4.7
Alismatis Rhizoma	MOL000359	Sitosterol	12303645	Ccl4	2X6L	-5.2
Poria cocos	MOL000279	Cerevisterol	10181133	Ccl4	2X6L	-5.39
Poria cocos	MOL000282	Ergosta-7,22E-dien-3beta-ol	5283628	Ccl4	2X6L	-5.42
Poria cocos	MOL000296	Hederagenin	73299	Ccl4	2X6L	-5.48
Alismatis Rhizoma	MOL000856	Alisol C monoacetate	14036813	Ccl4	2X6L	-5.49
Poria cocos	MOL000275	Trametenolic acid	125181708	Ccl4	2X6L	-5.5
Poria cocos	MOL000283	Ergosterol peroxide	5351516	Ccl4	2X6L	-5.67
Alismatis Rhizoma	MOL000849	16β-methoxyalisol B monoacetate	162953612	Ccl4	2X6L	-5.91
Alismatis Rhizoma	MOL000862	[(1S,3R)-1-[(2R)-3,3-dimethyloxiran-2-yl]-3-[(5R,8S,9S,10S,11S,14R)-11-hydroxy-4,4,8,10,14-pentamethyl-3-oxo-1,2,5,6,7,9,11,12,15,16-decahydrocyclopenta[a]phenanthren-17-yl]butyl] acetate	14036811	Ccl4	2X6L	-5.93
Poria cocos	MOL000273	(2R)-2-[(3S,5R,10S,13R,14R,16R,17R)-3,16-dihydroxy-4,4,10,13,14-pentamethyl-2,3,5,6,12,15,16,17-octahydro-1H-cyclopenta[a]phenanthren-17-yl]-6-methylhept-5-enoic acid	10743008	Ccl4	2X6L	-6.03

Alismatis Rhizoma	MOL000831	Alisol B monoacetate	163083573	Ccl4	2X6L	-6.83
Poria cocos	MOL000282	Ergosta-7,22E-dien-3beta-ol	5283628	Cd40	7P3I	-5.36
Poria cocos	MOL000275	Trametenolic acid	125181708	Cd40	7P3I	-5.55
Poria cocos	MOL000296	Hederagenin	73299	Cd40	7P3I	-5.74
Alismatis Rhizoma	MOL000849	16β-methoxyalisol B monoacetate	162953612	Cd40	7P3I	-5.78
Poria cocos	MOL000273	(2R)-2-[(3S,5R,10S,13R,14R,16R,17R)-3,16-dihydroxy-4,4,10,13,14-pentamethyl-2,3,5,6,12,15,16,17-octahydro-1H-cyclopenta[a]phenanthren-17-yl]-6-methylhept-5-enoic acid	10743008	Cd40	7P3I	-5.81
Poria cocos	MOL000279	Cerevisterol	10181133	Cd40	7P3I	-5.84
Alismatis Rhizoma	MOL000853	Alisol B	15558620	Cd40	7P3I	-5.92
Alismatis Rhizoma	MOL002464	1-Monolinolein	6436630	Cd40	7P3I	-5.94
Alismatis Rhizoma	MOL000359	Sitosterol	12303645	Cd40	7P3I	-5.97
Poria cocos	MOL000283	Ergosterol peroxide	5351516	Cd40	7P3I	-6.23
Alismatis Rhizoma	MOL000831	Alisol B monoacetate	163083573	Cd40	7P3I	-6.68
Alismatis Rhizoma	MOL000862	[(1S,3R)-1-[(2R)-3,3-dimethyloxiran-2-yl]-3-[(5R,8S,9S,10S,11S,14R)-11-hydroxy-4,4,8,10,14-pentamethyl-3-oxo-1,2,5,6,7,9,11,12,15,16-decahydrocyclopenta[a]phenanthren-17-yl]butyl] acetate	14036811	Cd40	7P3I	-6.85
Alismatis Rhizoma	MOL000856	Alisol C monoacetate	14036813	Cd40	7P3I	-7.29
Poria cocos	MOL000273	(2R)-2-[(3S,5R,10S,13R,14R,16R,17R)-3,16-dihydroxy-4,4,10,13,14-pentamethyl-2,3,5,6,12,15,16,17-octahydro-1H-cyclopenta[a]phenanthren-17-yl]-6-methylhept-5-enoic acid	10743008	Il-1β	5R8Q	-6.16
Poria cocos	MOL000296	Hederagenin	73299	Il-1β	5R8Q	-6.64
Poria cocos	MOL000282	Ergosta-7,22E-dien-3beta-ol	5283628	Il-1β	5R8Q	-6.89
Alismatis Rhizoma	MOL000849	16β-methoxyalisol B monoacetate	162953612	Il-1β	5R8Q	-7.13
Alismatis Rhizoma	MOL000862	[(1S,3R)-1-[(2R)-3,3-dimethyloxiran-2-yl]-3-[(5R,8S,9S,10S,11S,14R)-11-hydroxy-4,4,8,10,14-pentamethyl-3-oxo-1,2,5,6,7,9,11,12,15,16-	14036811	Il-1β	5R8Q	-7.31

		decahydrocyclopenta[a]phenanthren-17-yl]butyl] acetate				
Poria cocos	MOL000275	Trametenolic acid	125181708	Il-1β	5R8Q	-7.32
Alismatis Rhizoma	MOL002464	1-Monolinolein	6436630	Il-1β	5R8Q	-7.36
Poria cocos	MOL000279	Cerevisterol	10181133	Il-1β	5R8Q	-7.58
Alismatis Rhizoma	MOL000853	Alisol B	15558620	Il-1β	5R8Q	-7.64
Alismatis Rhizoma	MOL000831	Alisol B monoacetate	163083573	Il-1β	5R8Q	-7.65
Alismatis Rhizoma	MOL000359	Sitosterol	12303645	Il-1β	5R8Q	-7.87
Alismatis Rhizoma	MOL000856	Alisol C monoacetate	14036813	Il-1β	5R8Q	-8.33
Poria cocos	MOL000283	Ergosterol peroxide	5351516	Il-1β	5R8Q	-8.49
